# Distribution and altitudinal patterns of carbon and nitrogen storage in various forest ecosystems in the central Yunnan Plateau, China

**DOI:** 10.1038/s41598-021-85710-8

**Published:** 2021-03-18

**Authors:** Jianqiang Li, Qibo Chen, Zhuang Li, Bangxiao Peng, Jianlong Zhang, Xuexia Xing, Binyang Zhao, Denghui Song

**Affiliations:** School of Ecology and Environment, Southwest Forestry University, Kunming, 650224 China

**Keywords:** Ecology, Plant sciences

## Abstract

The carbon (C) pool in forest ecosystems plays a long-term and sustained role in mitigating the impacts of global warming, and the sequestration of C is closely linked to the nitrogen (N) cycle. Accurate estimates C and N storage (S_C_, S_N_) of forest can improve our understanding of C and N cycles and help develop sustainable forest management policies in the content of climate change. In this study, the S_C_ and S_N_ of various forest ecosystems dominated respectively by *Castanopsis carlesii and Lithocarpus mairei* (EB), *Pinus yunnanensis* (PY), *Pinus armandii* (PA), *Keteleeria evelyniana* (KE), and *Quercus semecarpifolia* (QS) in the central Yunnan Plateau of China, were estimated on the basis of a field inventory to determine the distribution and altitudinal patterns of S_C_ and S_N_ among various forest ecosystems. The results showed that (1) the forest S_C_ ranged from 179.58 ± 20.57 t hm^−1^ in QS to 365.89 ± 35.03 t hm^−1^ in EB. Soil, living biomass and litter contributed an average of 64.73%, 31.72% and 2.86% to forest S_C_, respectively; (2) the forest S_N_ ranged from 4.47 ± 0.94 t ha^−1^ in PY to 8.91 ± 1.83 t ha^−1^ in PA. Soil, plants and litter contributed an average of 86.88%, 10.27% and 2.85% to forest S_N_, respectively; (3) the forest S_C_ and S_N_ decreased apparently with increasing altitude. The result demonstrates that changes in forest types can strongly affect the forest S_C_ and S_N_. This study provides baseline information for forestland managers regarding forest resource utilization and C management.

## Introduction

C and N are major constituents of plant and soil organic matter and play a fundamental role in nutrient cycling, plant growth, and ecological functions^1,2^. Forest S_C_ is the most important part of the global C pool across various terrestrial ecosystems and plays a long-term and continuous role in mitigating the effects of global warming^3–5^. N is a vital and limiting nutrient in forest ecosystems, and C storage is closely linked to the N cycle^6^. Furthermore, N deposition alters S_C_ and S_N_^7,8^. Consequently, accurate identification of the spatial patterns of forest S_C_ and S_N_ is important for accessing the global C and N pool.

Forest S_C_ is estimated to account for approximately 45% of terrestrial ecosystem S_C_^9,10^. In forest ecosystems, C is stored in living biomass, litter and soils^11–13^. Living biomass has a great capacity to sequester atmospheric C and the aboveground living biomass has been considered as a major C pool^14,15^. Soil is another indispensable component of forest ecosystems and acts as an important C pool in terrestrial ecosystems^16,17^. The amount of C stored in soil is approximately double the amount in the atmosphere^17,18^. Consequently, exploring the distribution patterns of S_N_ in forest ecosystems is essential for understanding the C cycle. Many studies have explored the spatial distribution of S_C_ in forest ecosystems at a landscape scale using remote sensing and statistical methods^4,12,16,19,20^; however, these estimates are not reliable in hilly terrain, because the mountainous and hilly conditions can increase errors of forest vertical structure measured using remote sensors^13^. Hence, to accurately quantify forest S_C_ at a large scale, it is essential to develop estimates based on ground measurements. Forest inventory data are recognized as one of the most reliable sources of data for global C cycle research^4^.

The amount of C stored in forest vegetation and soil is considered to be the result of a long-term balance between C absorption and release^20,21^. The magnitude of S_C_ and S_N_ in forests depends on stand age, species composition, climate variability, geographical circumstances, management strategy and natural disturbances^22–24^. The distribution patterns of S_C_ and S_N_ also differ among spatial landscape patterns, plant species and plant organs^25^. Mopan Mountain in the central Yunnan Plateau is located in the Yunnan-Guizhou Plateau and the southern margin of the Qinghai-Tibet Plateau. The area belongs to a subtropical mountain climate region^26^, and vegetation patterns shift vertically due to changes in altitude. The main forest vegetation types are subtropical evergreen broad-leaved forest, subtropical mixed coniferous and broad-leaved forests, coniferous forest and alpine forest. In this region, forests cover more than 72.6% of the land area, and they represent the most important forest resources in the central Yunnan Plateau and in Yunnan Province. The main objectives of this study are to (1) assess the spatial variation in forest biomass based on a field inventory; (2) characterize the spatial variation in C and N density and storage in forest ecosystems; and (3) explore the impact of altitude on biomass and S_N_ and S_C_ in Mopan Mountain. This study will provide baseline information for forestland managers regarding forest resource utilization and C and N management.

## Materials and methods

### Study area

This study was conducted in Mopan Mountain National Forest Park (23°46′18″N–23°54′34″N, 101°16′06″E–101°16′12″E) in the central Yunnan Plateau of Yunnan province, southwestern China. The total area of the forest is about 7348.5 ha with an altitude from 1260 to 2614 m a.s.l.

The area belongs to a subtropical mountain climate region. The temperature ranges from – 2.2 to 33 °C with a mean annual temperature 15 °C, and the annual rainfall is approximately 1050 mm. Precipitation shows strong seasonal variation with approximately 85% occurring in the rainy season from May to October and the left 15% occurring in the dry season from November to the next April^26^.

The study sites were occupied by subtropical evergreen broad-leaved forest, coniferous forest and alpine forest dominated respectively by *C. carlesii* and *Lithocarpus mairei* (EB), *Pinus yunnanensis* (PY), *Pinus armandii* (PA), *Keteleeria evelyniana* (mixed with the *Quercus* species, KE) and *Quercus semecarpifolia* (QS). The characteristics of these forests are listed in Table 1.

**Table 1:** Stand information of various study forests in Mopan Mountain in the central Yunnan Plateau.

Forest type	Mean tree age/yr	Mean diameter at breast height/cm	Mean height/m	Wood density/ha	Main species composition		
					Tree	Shrub	Herb
PY	35	15.2	10.2	1887	*Pinus yunnanensis, Quercus aliena* Blume*, Schima superba* Gardn. et Champ.*, Pyrus pseudopashia* Yü	*Rhododendron spiciferum* Franch.*, Quercus variabilis* Blume*, Vaccinium bracteatum* Thunb	*Carex doisutepensis* Schreb.*, Heteropogon contortus* (Linn.) Beauv
PA	30	12.0	10.6	2029	*Pinus armandii* Franch., *Eurya obliquifolia* Hemsl., *Ternstroemia gymnanthera* (Wight et Arn.) Beddome	*Vaccinium duclouxii* (Levl.) Hand.-Mazz.*, Cyclobalanopsis glauca* (Thunberg) Oersted*, Ternstroemia gymnanthera* (Wight et Arn.) Beddome	*Ophiopogon bodinieri* Levl.*, Arthraxon hispidus* (Trin.) Makino
QS	40	5.9	3.3	3586	*Quercus semecarpifolia* Smit*h., Quercus fabri* Hance*, Ternstroemia gymnanthera* (Wight et Arn.) Beddome*, Lyonia ovalifolia* (Wall.) Drude	*Vaccinium bracteatum* Thunb., *Rhododendron moulmainense* Hook. f., *Gaultheria fragrantissima* Wall	*Gentiana cephalantha* Franch. ex Hemsl., *Smilax ferox* Wall. ex Kunth
KE	50	21.2	7.6	1475	*Keteleeria evelyniana* Mast., *Quercus acutissima* Carr., *Rhododendron delavay* Franch., *Rhododendron minutiflorum* Franch., *Quercus aliena* Blume	*Jasminum grandiflorum* L.*, Myrica esculenta* Buch.-Ham., *Rhododendron minutiflorum* Hu	*Ageratina adenophora* (Sprengel) R. M. King & H. Robinson*, Cyperus glomeratus* Nees
EB	60	9.7	11.1	3085	*Castanopsis carlesii* (Hemsl.) Hayata., *Lithocarpus mairei* (Schottky) Rehder, *Camellia mairei* (Levl.) Melch., *Dichotomanthes tristaniicarpa* Kurz*, Rhododendron delavayi* Franch., *Vaccinium bracteatum* Thunb., *Betula utilis* D. Don	*Camellia mairei* (Levl.) Melch., *Lithocarpus mairei* (Schottky) Rehder*, Vaccinium bracteatum* Thunb., *Rhododendron spiciferum* Franch., *Eurya yunnanensis* Hsu, *Symplocos anomala* Brand, *Ternstroemia gymnanthera* (Wight et Arn.) Beddome	*Indocalamus longiauritus* Handel-Mazzetti, *Ophiopogon bodinieri* Levl

### Study design and sampling

#### Study design

To test the variation in S_C_ and S_N_ among various forests, 16 sample plots in each forest were chosen for analysis. These 16 sample plots contained one 100 m × 100 m and fifteen 30 m × 30 m tree plots, and each tree plot had three shrub and three herb plots. The sizes of the shrub and herb plots were 5 m × 5 m and 1 m × 1 m, respectively^17^. The sample plots were distributed across the altitude range as follows: for QS, from 2467 to 2611 m; for PY, from 2012 to 2151 m; for PA, from 2035 to 2381 m; for KE, from 1865 to 2265 m; and for EB, from 1450 to 2436 m.

#### Plant census and sampling

In each tree plot, census of plant individuals which diameter at breast height was more than 1 cm was performed. In addition, in each shrub and herb plot, the species name and abundance were recorded^2,17,27^. All plant individuals in each plot were collected with different parts for C and N testing, i.e., trees with roots, trunks, leaves, branches and bark; shrubs with roots, stems and leaves; and herbs with above- and belowground part.

#### Litter sampling

Triplicate plots with a size of 1 m × 1 m were established in the tree plots for ground litter sampling^28^. For each of these samples, horizons L, F and H were separated and carefully placed in plastic bags for determining of the dry weight and C and N contents. The L horizon was composed of fresh or slightly discoloured material that was not weak or friable; the F horizon was composed of medium to strongly fragmented material with many mycelia and thin roots; and the H horizon consisted of humified amorphous material.

#### Mineral soil sampling

Mineral soil samples were collected from each tree plot, with three replicates. Most of the slope gradients of these soil profiles were less than 15°. After removal of the forest floor mass, soil samples were collected from three layers: 0–20 cm, 20–40 cm and 40–60 cm, and the corresponding soil bulk density (BD) of each layer was measured using the cutting-ring method^29^. The soil samples were placed in sacks and air dried for soil C and N testing.

#### Laboratory analysis

Shrub, herb and ground litter samples were dried to a constant weight at 105 °C and then weighed for biomass estimation. Plant and soil total N concentrations were determined by a continuous flow analytical system (Analytical AA3, SEAL, Germany) with sulfuric acid (H_2_SO_4_) and hydrogen peroxide (H_2_O_2_) digestion^30^. The total C concentration was determined by an elemental analyser (Vario TOC cube, Elementar, Germany)^31^.

### The estimation of biomass, S_C_, and S_N_

#### The estimation of forest vegetation biomass

Tree biomass (roots, trunks, leaves, branches and bark biomass) was estimated using allometric equations based on long-term practical measurements of forest vegetation in southwestern China^32–38^. The shrub and herb biomass was directly expressed as their dry weights. For each forest plot, the total biomass was the sum of the biomass of each vegetation type in the plot.

#### The estimation of plant, litter and soil S_C_ and S_N_

The S_C_ (t ha^−1^) and S_N_ (t ha^−1^) of trees, shrubs, and herbs were obtained by multiplying the forest vegetation biomass (t ha^−1^) by the corresponding C and N content coefficient^17,29^.

The litter S_C_ and S_N_ were the sum of the S_C_ and S_N_ of horizons L, F and H. The litter S_C_ and S_N_ storage was calculated by the following formula^17^:

1 $$ Litter\;S_{C} = \mathop \sum \limits_{i = 1}^{n} LB_{i} \times TC_{i} ;\;\;\;Litter\;S_{N} = \mathop \sum \limits_{i = 1}^{n} LB_{i} \times TN_{i} $$

where S_C_ (t ha^−1^) and S_N_ (t ha^−1^) are the respective litter C and N storage; $TC_{i}$ and $TN_{i}$ are the C and N (g kg^−1^) contents of horizons L, F and H, respectively; and $LB_{i}$ is the litter biomass (dry litter weight) of horizons L, F and H.

The soil S_C_ and S_N_ were calculated as the sum of the S_C_ and S_N_ of the 0–20 cm, 20–40 cm and 40–60 cm soil layers. The soil S_C_ and S_N_ were calculated using the following formula^21,39^:

2 $$ Soil \;S_{C} = \mathop \sum \limits_{i = 1}^{n} BD_{i} \times TC_{i} \times D_{i} ;\;\;\;Soil\;S_{N} = \mathop \sum \limits_{i = 1}^{n} BD_{i} \times TN_{i} \times D_{i} $$

where S_C_ and S_N_ are soil total C storage (t ha^−1^) and N storage (t ha^−1^), respectively, $BD_{i}$ is the soil BD (g cm^-3^), $TC_{i}$ and $TN_{i}$ are the soil total C and N contents (g kg^−1^), respectively, and $D_{i}$ is the soil layer thickness (cm).

#### Statistical analysis

Statistical analyses were carried out using the software Statistical Package for the Social Sciences 19 (SPSS 19) and Microsoft Office Excel (version 2013). One-way ANOVA was used to test whether the variations in S_C_ and S_N_ were significantly different among the plant, litter and soil and forest type components. Duncan’s shortest range test was used to examine the difference among different forest types at *P* < *0.05*. The relationships between altitude and biomass, S_C_ and S_N_ were examined by linear regression.

## Results

### Biomass in forest ecosystems

**Figure 1:**
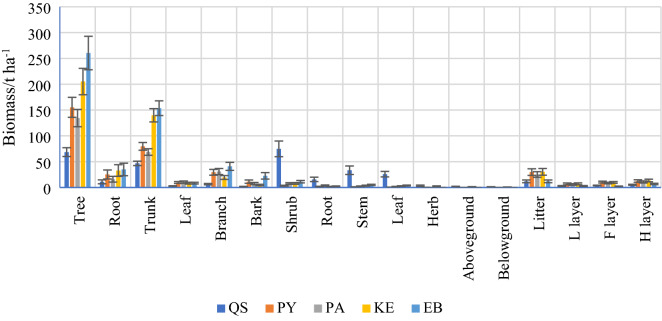
Biomass (t ha^−1^) allocation among plants and litter layer in various forests in the central Yunnan Plateau. Error bars mean standard deviation.

The biomass of the forest ecosystems in the central Yunnan Plateau ranged from 142.36 ± 18.36 to 271.77 ± 34.71 t ha^−1^. The biomass of the forest ecosystems was significantly different among the various forests (Table 2). Plant biomass made a significant contribution to ecosystem biomass and accounted for a much higher proportion (more than 90%) than forest litter. Tree biomass was significantly higher than that of shrubs and herbs in PY, PA, KE and EB and accounted for 99.64%, 94.46%, 95.33% and 95.88% of the total plant biomass, respectively. The tree and shrub biomass in QS accounted for a nearly equal proportion of plant biomass at 46.72% and 51.01%, respectively. The biomass of each component of plants and litter is presented in Fig. 1.

**Table 2:** Biomass (t ha^−1^) and proportion (%) of plant components and the litter layer in various forests in the central Yunnan Plateau.

	QS	PY	PA	KE	EB
Plant	146.56 ± 24.97 D	158.99 ± 2013 C	142.36 ± 18.36 D	215.30 ± 27.95 B	271.77 ± 34.71 A
Tree	68.47 ± 8.52 Da	155.24 ± 19.32 Ca	134.47 ± 16.73 Ca	205.25 ± 25.54 Ba	260.57 ± 32.42 Aa
Tree/Plant%	46.72	97.64	94.46	95.33	95.88
Shrub	74.76 ± 15.23 Aa	3.45 ± 0.70 Db	7.75 ± 1.58 Cb	7.81 ± 1.59 Cb	11.19 ± 2.28 Bb
Shrub/Plant%	51.01	2.17	5.44	3.63	4.12
Herb	3.33 ± 1.22 Ab	0.30 ± 0.11 Cc	0.14 ± 0.05 Cc	2.24 ± 0.82 Bc	0.01 ± 0.00 Dc
Her/plant%	2.27	0.19	0.10	1.04	0.00
Litter	11.92 ± 2.40B	29.81 ± 5.96 A	25.15 ± 5.02 A	30.28 ± 6.07 A	11.91 ± 2.43 B
L layer	2.92 ± 0.78 Ba	6.92 ± 1.85 Aab	5.73 ± 1.53 Ac	7.01 ± 1.87 Ac	2.91 ± 0.78 Bb
L layer/Litter%	24.5	23.21	22.78	23.15	24.43
F layer	3.84 ± 0.65 Ba	10.44 ± 1.77 Aa	8.61 ± 1.46 Ab	9.81 ± 1.66 Ab	2.25 ± 0.38 Bb
F layer/Litter%	32.21	35.02	34.23	32.40	18.89
H layer	5.15 ± 0.97 Ba	12.45 ± 2.34 Aa	10.80 ± 2.03 Aa	13.46 ± 2.54 Aa	6.75 ± 1.27 Ba
H layer/Litter%	11.92	29.81	25.15	30.28	11.91
Ecosystem	158.47 ± 27.37 D	188.80 ± 26.09 B	167.50 ± 23.38 C	245.58 ± 34.02 A	283.68 ± 37.14 A
Plant/Ecosystem%	94.22	91.43	91.69	92.28	96.79
Litter/Ecosystem%	5.78	8.57	8.31	7.72	3.21

Mean values ± standard deviations are illustrated; Different lowercase letters in each row indicate significant differences (*P* < *0.05*) among the plant components and litter layer, and different capital letters in each line indicate significant differences (*P* < *0.05*) among forests.

### C and N concentrations

**Figure 2:**
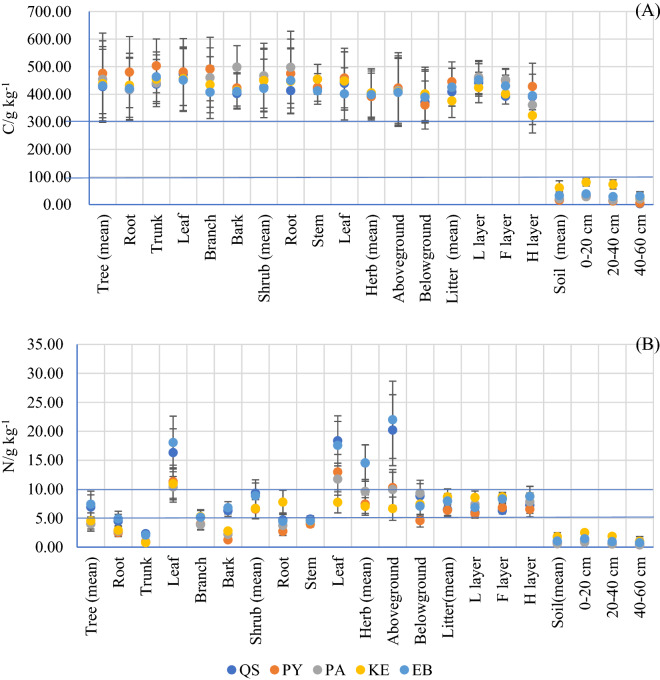
C (g kg^−1^) and N (g kg^−1^) concentrations in the plant components, litter layer and soil in various forests in the central Yunnan Plateau. Error bars mean standard deviation.

The C and N concentrations in plants, shrubs and herbs varied significantly among forests and their components (Fig. 2 A-B). Generally, the N concentration was classified into three levels by the lines in the figure. The N concentration was highest in the leaves of trees and shrubs and the aboveground parts of herbs, and it ranged from 6.64 ± 2.01 to 21.99 ± 6.66 g·kg^−1^. The N concentration in tree branches, shrub stems and the L, F and H litter horizons ranged from 3.86 ± 0.90 to 8.78 ± 1.73 g·kg^−1^, and these values were higher than those in the roots and trunks of trees, shrub roots and soil, which had N concentrations lower than 4.89 ± 1.31 g·kg^−1^. Significant differences were not observed in the C concentrations in the plant and litter components among different forests, which ranged from 323.21 ± 63.58 to 503.00 ± 97.56 g·kg^−1^, and the mean C concentration of the forest vegetation and litter was 425.80 ± 100.34 g·kg^−1^. However, the soil C concentrations were significantly lower than those in the plants and litter, i.e., less than 81.08 ± 13.62 g kg^−1^, with a mean of 29.74 ± 12.20 g·kg^−1^.

### S_C_ and S_N_

**Figure 3:**
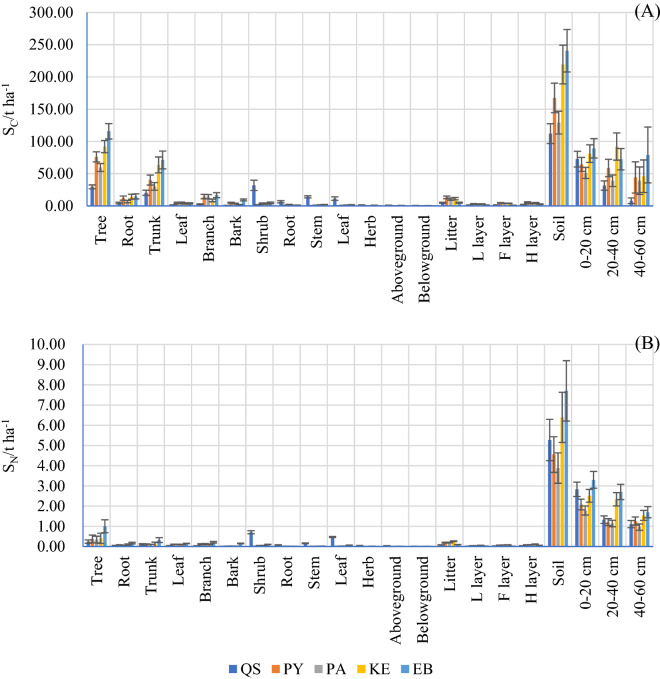
S_C_ (t ha^−1^) and S_N_ (t ha^−1^) storage allocation among the plant components, litter and soil layers in various forest types in the central Yunnan Plateau. Error bars mean standard deviation.

The ecosystem S_C_ was calculated as the sum of the plant S_C_, litter S_C_ and soil S_C_. The ecosystem S_C_ was significantly different among the forests (Table 3 and Fig. 3 A). The ecosystem S_C_ ranged from a high of 365.89 ± 35.03 t hm^−1^ in EB to a low of 179.58 ± 20.57 t hm^−1^ in QS. The levels of ecosystem S_C_ in PY, PA and KE were 258.38 ± 24.92, 203.01 ± 19.79 and 326.89 ± 31.71 t hm^−1^, respectively. The soil S_C_ contributed 62.40–67.06% to the ecosystem S_C_ in various forests and was higher than the contributions of plants and litter. Plant S_C_ accounted for 29.50–34.91%, and litter S_C_ accounted for only 1.35–5.15% of ecosystem S_C_.

**Table 3:** S_C_, (t ha^−1^), S_N_, (t ha^−1^) and their proportions (%) in the plants, litter and soil in various forest types in the central Yunnan Plateau.

	QS	PY	PA	KE	EB
Plant S_C_	62.70 ± 11.33 Db	77.76 ± 8.20 Cb	63.36 ± 7.04 Db	96.43 ± 10.49Bb	120.35 ± 13.01 Ab
Plant/Ecosystem%	34.91	30.09	31.21	29.50	32.89
Litter S_C_	4.82 ± 0.77 Cc	13.20 ± 2.12 Ac	10.45 ± 1.68 Bb	10.99 ± 1.81 Bc	4.85 ± 0.80 Cc
litter/Ecosystem%	2.68	5.11	5.15	3.44	1.35
Soil S_C_	112.06 ± 15.32 Da	164.42 ± 22.90 Ca	129.20 ± 17.67 Ca	219.21 ± 29.98 Ba	240.59 ± 32.90 Aa
Soil/Ecosystem%	62.40	64.80	63.64	67.06	65.76
Ecosystem S_C_	179.58 ± 20.57 D	258.38 ± 24.92 B	203.01 ± 19.79C	326.89 ± 31.71 A	365.89 ± 35.03A
Plant S_N_	1.01 ± 0.16 Ab	0.40 ± 0.19 Cb	0.39 ± 0.17 Cb	0.47 ± 0.26 Bb	1.11 ± 0.33 Ab
Plant/Ecosystem%	15.84	7.71	8.73	6.63	12.43
Litter S_N_	0.08 ± 0.01 Dc	0.19 ± 0.02 Bc	0.20 ± 0.02 Bc	0.26 ± 0.02 Ac	0.10 ± 0.01 Cc
litter/Ecosystem%	1.25	3.74	4.48	3.70	1.10
Soil S_N_	5.27 ± 1.02 Ca	4.55 ± 0.88 Da	3.88 ± 0.75 Ea	6.39 ± 1.24 Ba	7.70 ± 1.49Aa
Soil/Ecosystem%	82.92	88.55	86.79	89.67	86.48
Ecosystem S_N_	6.36 ± 1.19 C	5.14 ± 1.10 D	4.47 ± 0.94 D	7.13 ± 1.52 B	8.91 ± 1.83 A

Mean values ± standard deviations are illustrated; Different lowercase letters in each row indicate significant differences (*P* < *0.05*) among plant components and the litter layer, and different lowercase letters in each line indicate significant differences (*P* < *0.05*) among forest types.

The plant S_C_ of different forests varied significantly from 62.70 ± 11.33 t hm^−1^ in QS to 120.35 ± 13.01 t·hm^−1^ in EB, although the difference between QS and PA was not significant. Tree S_C_ contributed more than 94% to the plant S_C_ in PY, PA, KE and EB; however, the tree S_C_ of QS contributed only 47.11% to the plant S_C_. The shrub S_C_ of QS accounted for a high proportion of 50.78% of the plant S_C_, whereas the shrub and herb S_C_ in the other four forests contributed less than 1% to the plant S_C._ The litter S_C_ varied from a high concentration of 13.20 ± 2.12 t hm^−1^ in PY to a low concentration of 4.82 ± 0.77 t·hm^−1^ in QS. Generally, the S_C_ of different layers among the forests decreased in the order of H > F > L, while the litter S_C_ in EB decreased in the order of H > L > F. The highest soil S_C_ was in EB at 240.59 ± 32.90 t hm^−1^. The soil S_C_ in KE was 219.21 ± 29.98 t hm^−1^, which was significantly lower than that in EB but significantly higher than that in PY and PA, which were 164.42 ± 22.90 t hm^−1^ and 129.20 ± 17.67 t hm^−1^, respectively. The lowest soil S_C_ was in QS at 112.06 ± 15.32 t hm^−1^. In KE, the S_C_ at 20–40 cm was higher than that at 0–20 cm and the soil S_C_ decreased with increasing soil depth.

The S_N_ of the forest ecosystems varied significantly among the forests, although significant differences were not found between PY and PA (Table 3 and Fig. 3 B). The Ecosystem S_N_ ranged from 8.91 ± 1.83 t·ha^−1^ in EB to 4.47 ± 0.94 t·ha^−1^ in PA, and the S_N_ in KE, QS and PY was 7.13 ± 1.52 t·ha^−1^, 6.36 ± 1.19 t ha^−1^ and 5.14 ± 1.10 t ha^−1^_,_ respectively. Soil was the most important contributor to total S_N_ in the forest ecosystems and accounted for an average of 86.88% of Eco S_N_. Plant contributions to Eco S_N_ ranged from a high concentration of 15.83% in QS to a low concentration of 6.63% in KE, and litter only contributed an average of 2.85%.

The plant S_N_ differed significantly among the forests with different species and ranged from a high concentration of 1.11 ± 0.33 t·ha^−1^ in EB to a low concentration of 0.39 ± 0.17 t·ha^−1^ in PA. The tree S_N_ of PY, PA, KE and EB accounted for more than 85% of the living biomass S_N_, and the shrubs and herbs contributed less than 15%. However, the shrubs in QS stored more N than trees and the S_N_ of shrubs and trees contributed 24.42% and 70.36% to the living biomass S_N_, respectively. The estimated mean S_N_ of forest litter was 0.17 ± 0.01 t·ha^−1^, and the H layer stored approximately half of the litter N. The soil is a large N pool in forest ecosystems, and in this study, the soil S_N_ accounted for 86.88% on average of Eco S_N_. More S_N_ was stored in the topsoil (0–20 cm), with a contribution of 53.69%, 45.71%, 45.87%, 39.27% and 42.85% to the total soil (0–60 cm) S_N_ in QS, PY, PA, KE and EB, respectively.

### Correlation analysis of biomass, S_C_ and S_N_ in forest ecosystems and altitude

**Figure 4:**
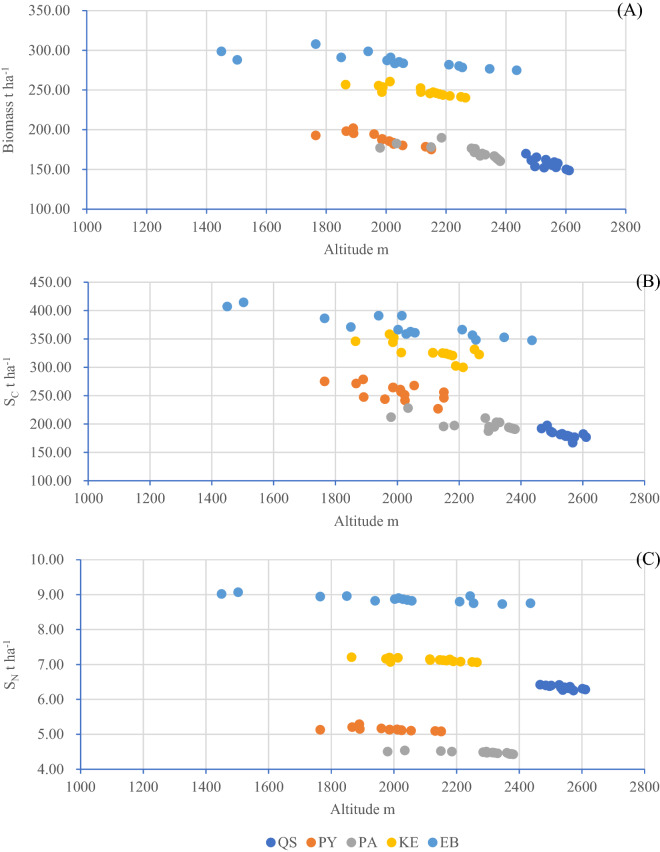
Altitudinal patterns of biomass, S_C_ and S_N_ of different forests in the central Yunnan Plateau.

The generalized linear model illustrates the effects of altitude on biomass, S_C_ and S_N_ of forest ecosystems, which decreased with increasing altitude (Fig. 4A-C). Whether calculated within the same forest or across all forests, significant correlations were found between the altitude and biomass at the *P* < *0.005* level (Table 4). The S_C_ of QS, PA, KE and EB was also significantly (*P* < *0.005*) correlated with altitude, but for PY, the correlation between S_C_ and altitude was significant at the *P* < *0.05* level. In all forests, S_C_ decreased significantly (*P* < *0.001*) with increasing altitude. A significant correlation (*P* < *0.005*) was observed between S_N_ and altitude in QS, PA, KE and EB, and a less significant correlation was observed between S_N_ and altitude (*P* < *0.01)* in PY. However, for all forests, the variation in S_N_ was not significant (*P* = *0.400*) with respect to altitude.

**Table 4:** The results of the generalized linear model analyses of the effects of altitude on biomass, S_C_ and S_N_. Three linear models were built: model I: Biomass (t ha^−1^) = C + a × AL (altitude, m); model II: S_C_ (t ha^−1^) = C + a × AL (altitude, m); and model III: S_N_ (t ha^−1^) = C + a × AL (altitude, m), where C is the regression constant and a is the regression coefficient of the given variable.

	C	a	R^2^	P
AL and Biomass				
QS	423.917	−0.105	0.558	0.001
PY	316.777	−0.065	0.767	0.000
PA	283.243	−0.049	0.566	0.001
KE	342.446	−0.045	0.733	0.000
EB	336.079	−0.024	0.569	0.001
All	468.772	−0.118	0.362	0.000
AL and S_C_				
QS	513.472	−0.131	0.558	0.001
PY	411.679	−0.078	0.356	0.019
PA	336.246	−0.060	0.474	0.003
KE	545.976	−0.103	0.547	0.002
EB	505.896	−0.067	0.786	0.000
All	713.268	−0.204	0.481	0.000
AL and S_N_				
QS	8.945	−0.0010	0.605	0.001
PY	5.767	−0.0003	0.421	0.009
PA	4.959	−0.0002	0.681	0.000
KE	7.783	−0.0003	0.557	0.010
EB	9.478	−0.0003	0.718	0.000
All	9.565	−0.0014	0.057	0.400

## Discussion

Different forest ecosystems have different C sequestration capacities. The total S_C_ values of the forest ecosystems of KE and EB in Mopan Mountain in the central Yunnan Plateau are apparently higher than the average values of forest ecosystems (258.83 t C∙ha^−1^) across China^27,40^, whereas the total S_C_ values of the forest ecosystems of QS PA and PY are lower. The results of the present research show that changes in forest types can strongly affect S_C_ and S_N_ values. Generally, broad-leaved species can store more C and N than conifers^30,41^. Although the alpine forest (QS) had the lowest S_C_, its S_N_ was higher than that in PY and PA. The S_C_ and S_N_ of forests in Mopan Mountain in the central Yunnan Plateau averaged 266.75 ± 26.40 t ha^−1^ and 6.40 ± 1.32 t ha^−1^, respectively_._ With respect to S_C_, the living biomass, litter layer and soil accounted for 31.72, 3.55% and 64.73% of the total C storage, respectively. The corresponding S_N_ accounted for 10.27%, 2.85% and 86.88% of the total N storage, respectively. The current and previous results indicate that the soil is the most important component for S_C_ and S_N_ in forest ecosystems^42,43^.

The living biomass of forests is one of the major C and N pools. Quantification of stored C in the living biomass of a forest is necessary for future management ^44^. The estimated mean living biomass S_C_ in this study was 84.12 t·ha^−1^, which is much higher than the average values of vegetation C storage in Chinese forest ecosystems (57.07 t ha^−1^)^45,46^. This finding is mainly because of the high tree density and low anthropogenic disturbance at the location of Mopan Mountain National Forest Park. The tree growth rate and biomass allocation to different tree parts and varying rates of C sequestration in ecosystem components can affect the rate of C sequestration and longevity of C storage^2,41^. The present study showed that the S_C_ in plants ranged from 62.70 ± 11.33 t·ha^−1^ in QS to 120.35 ± 13.01 t·ha^−1^ in EB, which accounted for 31.72% of the total C storage. Among all forests, QS had the lowest S_C_ in living biomass, which was caused by its lower biomass and lower C concentration in living biomass. However, the higher biomass in EB resulted in higher S_C_ of living biomass compared with the other groups in Mopan Mountain in the central Yunnan Plateau. The S_N_ in living biomass varied from a high of 1.11 ± 0.33 t·ha^−1^ in EB to a low of 0.39 ± 0.17 t·ha^−1^ in PA, with a mean contribution of 10.27% to total S_N_.Tree S_C_ and S_N_ accounted for a large proportion of living biomass S_C_ and S_N_ in PY, PA, KE and EB, whereas shrubs contributed more C and N than trees to living biomass S_C_ and S_N_ in QS. The S_C_ and S_N_ of vegetation are mainly determined by the biomass of live vegetation components and C and N contents. Consequently, the interspecific differences in tree biomass caused by inherent variation in growth rates^47–49^ were the main reasons for the variations in S_C_ and S_N_ allocation among forests. Furthermore, the effect of forest species on the growth and diversity of understorey plant biomass^2,30,50^ also resulted in the variation in S_C_ and S_N_ allocation in forest vegetation.

Forest litter and its decomposition rate are key factors in nutrient cycling in forest ecosystems^51^, and the current litter S_C_ in the world’s forests is estimated at 43 ± 3 Pg·C (5% of total forest C)^52^. In the present study, the estimated mean litter S_C_ and S_N_ in the forests were 8.93 ± 1.44 t ha^−1^ and 0.17 ± 0.01 t ha^−1^, which accounted for 3.55% and 2.85% of the total S_C_ and S_N_, respectively. The mean litter S_C_ in this study is slightly higher than the mean litter S_C_ in China (8.21 t·ha^−1^)^49^. The study also found that conifer litter stored more C and N than broadleaf litter, and a similar result was found in previous studies^41,53^. The above results occurred mainly because conifer litter is more difficult to decompose than broadleaf litter, resulting in a higher rate of litter accumulation on the forest floor.

The estimated mean soil S_C_ and S_N_ of different forests in this study were 173.70 ± 23.75 t·ha^−1^ and 5.56 ± 1.08 t·ha^−1^, which accounted for 64.73% and 86.88%, respectively, of the total S_C_ and S_N_. The results showed that soil is the largest C pool in forest ecosystems, similar to a previous study conducted in China^2,30,42^. The mean reported value of soil S_C_ was 193.55 t ha^−1^ in Chinese forest ecosystems ^45,46^, and the soil S_N_ was 6.27 t ha^−1^ in subtropical forests of China^54^. The S_C_ and S_N_ of KE and EB was higher and that of the other forests in this study was lower than the mean soil S_N_ in China and soil S_N_ in subtropical forests in China. The C stored in soil is significantly influenced by the C inputs (e.g., litter decomposition) and soil organic matter decomposition^55^. Therefore, S_C_ is determined by the balance between the input or output patterns and controlled mainly by tree species under similar environmental conditions^17,41^. There were significant differences in the soil S_C_ and S_N_ at depths of 0–20 cm, 20–40 cm and 40–60 cm among the forests. The topsoil (0–20 cm) in the forests stored 43.38% of the C and 45.48% of the N from 0 to 60 cm. The soil C and N were mainly stored in the topsoil^42,43,56^, which is probably because of the variation in the soil bulk density and concentrations of C and N in soil layers, which are two important determining factors of S_C_ and S_N_ at fixed soil depths ^17,57^. Although the soil bulk density decreased with increasing soil depth, the topsoil contained more C and N.

The forest ecosystem biomass (158.47 ± 27.37 to 283.68 ± 37.14 t·ha^−1^, with an average of 208.81 ± 29.60 t ha^−1^), S_C_ (179.58 ± 20.57 to 365.89 ± 35.03 t·ha^−1^, with an average of 266.75 ± 26.40 t ha^−1^) and SN (4.47 ± 0.94 to 8.91 ± 1.83 t ha^−1^ with an average of 6.40 ± 1.32 t ha^−1^) in the five forests in Mopan Mountain decreased with increasing altitude, although the S_C_ of PY and the S_N_ of all forest ecosystems in this study were not highly significantly correlated with altitude. Previous reports indicated that the soil S_C_ in forest ecosystems increases with altitude^58,59^ and the living biomass and total S_C_ of forest ecosystems decreased significantly with increasing latitude in different regions^60,61^ because increasing altitude changed the climate factors (i.e., temperature and precipitation) and resulted in the shifting of vegetation types and a decline in net primary production and litterfall^58,62,63^. The vegetation patterns in the study area shifted vertically due to changes in altitude. With increasing altitude, the forest vegetation types in this area shifted from subtropical evergreen broad-leaved forest, subtropical mixed coniferous and broad-leaved forest, and coniferous forest to alpine forest, and the living biomass of the forests declined significantly. Therefore, the total S_C_ and S_N_ of forest ecosystems exhibited decreasing trends with increasing altitude.
